# Neonatal Ovarian Cysts with Torsion: Correlation Between Preoperative Ultrasound Classification and Ovarian Preservation Outcomes

**DOI:** 10.3390/children13081092

**Published:** 2026-08-17

**Authors:** Xiaoli Chen, Shuangshuang Liu, Weiwei Chen, Zhigang Gao, Qingjiang Chen

**Affiliations:** 1 Department of General Surgery, The Children’s Hospital, Zhejiang University School of Medicine, National Clinical Research Center for Children and Adolescents’ Health and Diseases, 3333 Binsheng Rd, Hangzhou 310000, China; chenxiaoli2020@zju.edu.cn (X.C.); 11718199@zju.edu.cn (W.C.); ebwk@zju.edu.cn (Z.G.); 2 Department of Ultrasound, The Children’s Hospital, Zhejiang University School of Medicine, National Clinical Research Center for Children and Adolescents’ Health and Diseases, 3333 Binsheng Rd, Hangzhou 310000, China; 6514004@zju.edu.cn

**Keywords:** neonate, ovary torsion, surgery

## Abstract

**Highlights:**

**What are the main findings?**

• In neonatal ovarian cysts with surgically confirmed torsion, simple cysts had a 44.4% ovarian survival rate versus 0% for complex cysts (*p* < 0.001).
• Among the 9 torsed simple cysts, only 2 (22.2%) had preoperative sonographic torsion signs; the remaining 7 were operated on for size, and 4 (57.1%) had intraoperative necrosis.

**What are the implications of the main findings?**

• Preoperative ultrasound classification is associated with ovarian salvage. A simple cyst appearance does not exclude torsion, though our findings are limited to surgically confirmed cases.

**Abstract:**

**Objective**: We aimed to evaluate the association between preoperative ultrasound features, dynamic changes, and ovarian outcomes in neonatal ovarian torsion, and to compare ovarian survival across ultrasound subgroups. **Methods**: This retrospective study included 58 neonates with prenatally or postnatally diagnosed ovarian cysts who had surgically confirmed ovarian torsion and were treated at our institution between June 2015 and July 2025. **Results**: Of the 58 cases, 45 (77.6%) underwent oophorectomy and 13 (22.4%) underwent ovary-sparing surgery. Ovarian survival was achieved in 44.4% (4/9) of simple cysts compared with 0% (0/49) of complex cysts (RD +44.4%, 95% CI: +14.8% to +74.1%; *p* < 0.001). Among the 9 simple cysts, only 2 (22.2%) had preoperative sonographic signs of torsion; the other 7 underwent surgery due to size-related indications, of whom 4 (57.1%) had necrosis at surgery. In the 50 cases with serial follow-up, observation window, cyst size changes, morphological shifts, and age at surgery did not differ significantly between the oophorectomy and ovary-sparing surgery groups (all global *p* > 0.05). **Conclusions**: In neonatal ovarian cysts with torsion, the overall ovarian survival rate was low (6.9%, 4/58). However, a salvageable subgroup was identified: simple cysts had a 44.4% (4/9) survival rate, whereas complex cysts had 0% (0/49), indicating that ovarian salvage is rarely achievable in complex cysts. The conclusions are limited to surgically confirmed ovarian torsion cases and should not be generalized to other neonatal ovarian cysts.

## 1. Introduction

Neonatal ovarian cysts are generally regarded as benign ovarian lesions induced by maternal hormonal stimulation, and the vast majority resolve spontaneously within first year after birth [1,2]. Ovarian torsion represents a major complication of this disease, which may cause ovarian ischemia, necrosis, or autoamputation in severe cases [3]. Traditionally, neonatal ovarian cysts are categorized as either simple or complex according to their ultrasound features [4,5]. Simple cysts typically resolve spontaneously without intervention, whereas complex cysts are often associated with an unfavorable prognosis [6].

A major debate regarding contemporary treatment protocols concerns the choice between observation and surgical intervention for asymptomatic ovarian cysts. Most studies advocate conservative management for simple cysts measuring <4 cm [3,4,6,7]. Clinicians have not reached agreement on how to manage ovarian cysts that are >4 cm or demonstrate complex features on ultrasound [7,8,9,10]. The 2024 European Paediatric Surgeons’ Association guidelines [11] advise surgical treatment for neonatal simple ovarian cysts over 4 cm within 14 days of birth. Contemporary surgical practice recommends ovarian-sparing approaches whenever feasible, including detorsion of ischaemic-appearing ovaries. However, these suggestions rely predominantly on cyst size and underemphasize two essential concerns. For one thing, is there a possibility of silent torsion in cysts that appear simple under pre-operative sonography? For another, what is the likelihood of ovarian survival after torsion has occurred? Quantitative estimates of ovarian survival rates based on preoperative ultrasound appearance in surgically confirmed torsion cases remain limited. Notably, two distinct clinical questions exist: whether to operate on a neonatal ovarian cyst, and whether the ovary can be salvaged after torsion has occurred. This study addresses the latter—evaluating the association between preoperative ultrasound appearance and ovarian survival in surgically confirmed torsion cases.

This retrospective analysis included 58 neonates with surgically confirmed ovarian torsion. We systematically evaluated the correlations between preoperative ultrasonographic features, dynamic changes, timing of operation and ovarian outcomes, aiming to provide quantitative evidence for stratified clinical management of neonatal ovarian cysts. We hypothesized that preoperative ultrasound classification (simple vs. complex) is associated with ovarian survival. The primary outcome was ovarian survival, defined as the presence of surviving ipsilateral ovarian tissue on postoperative ultrasound (identifiable follicular structures and detectable blood flow).

## 2. Materials and Methods

This study was approved by the Ethics Committee of our center (2024-IRB-0176-P-02). This was a descriptive retrospective study. All analyses were exploratory in nature and no causal claims are made. All patients who received surgical intervention for ovarian cysts at our center between June 2015 and July 2025 were identified and enrolled for analysis. Inclusion criteria were as follows: 1. prenatal or postnatal diagnosis of ovarian cyst within the first month of life; 2. intraoperative confirmation of ovarian torsion; 3. availability of complete clinical and imaging data. Exclusion criteria included: 1. cases with intraoperative findings of ovarian autoamputation; 2. incomplete clinical or imaging data.

Collected data included patient demographics, preoperative ultrasound findings, surgical details, pathology reports, and follow-up information. Based on the classification systems proposed by Nussbaum et al. [5] and Kim et al. [12], seven sonographic indicators were defined: (1) internal low-level echoes—diffuse hazy echogenicity within the cyst lumen; (2) fluid–fluid or fluid–debris level—visible layering of echogenic sediment; (3) septation or reticular/mesh-like pattern—thin or thick echogenic strands traversing the cyst; (4) internal floating echoes—mobile punctate or flocculent echogenic materials; (5) intracystic calcification—echogenic foci with or without acoustic shadowing; (6) thickened or echogenic wall—abnormally thickened or brightly echogenic wall, with/without double-layered appearance, distinct from calcification; and (7) solid component—echogenic tissue protruding from the cyst wall. Cysts exhibiting none of these features were classified as simple, whereas those presenting one or more features were classified as complex. Ultrasound classification was performed by a senior radiologist with over 10 years of experience in pediatric ultrasound, who reviewed the original ultrasound images. The reviewer was blinded to the surgical and pathological outcomes. In cases where the reviewer’s interpretation differed from the original report, a consensus was reached through discussion with the original sonographer.

Preoperative ultrasound data covered the initial postnatal ultrasound scan and the last ultrasound examination before surgery. The ultrasound observation window was defined as the time interval between the first postnatal imaging and preoperative sonography. As our institution is a dedicated children’s hospital, all neonates were referred from other maternity units after birth, and prenatal ultrasound data were therefore unavailable. Eight neonates were admitted directly for surgery at initial presentation, without serial ultrasound monitoring. The remaining 50 neonates underwent longitudinal ultrasonographic follow-up, among whom the initial scan was performed within 2 weeks of life in 23 cases and beyond 2 weeks in 27 cases. Surgery was indicated for (1) ultrasound findings suggestive of torsion, (2) cysts measuring ≥4 cm in diameter, (3) progressive enlargement exceeding 4 cm during follow-up, or (4) symptoms resulting from compression of adjacent organs by the cyst.

Given that the primary endpoint of this study was ovarian survival, the follow-up protocol was tailored accordingly. For patients who underwent ovary-sparing surgery (*n* = 13), ovarian survival was assessed via serial ultrasound examinations (median follow-up 12.8 months, range: 6.4–57.7 months). Ipsilateral ovarian survival was defined as the presence of a visible ovary on the affected side with identifiable follicular structures and detectable blood flow on postoperative ultrasound. Cases without definitive visualization were classified as ovarian loss to maintain a conservative estimate. For those who underwent oophorectomy, the intraoperative findings served as the definitive endpoint for ovarian outcome.

Three patients had bilateral ovarian involvement. In all cases, torsion occurred on the side with the cyst. The contralateral ovary was managed by fenestration in two cases (simple cysts without torsion) and by detorsion in one case (isolated torsion without cyst formation). The contralateral findings were not included in the primary analysis. Stata 17.0 was adopted to carry out statistical calculations. Continuous data were reported as median (range) and categorical data as number (%). Comparisons across groups were performed using chi-square and Fisher’s exact tests. To account for multiple comparisons, Bonferroni correction was applied with an adjusted significance threshold of α = 0.05/19 = 0.0026 for the 19 comparisons performed in the subsequent results tables. Unadjusted *p*-values are reported for transparency; findings with *p* < 0.05 but ≥0.0026 are interpreted with caution and considered exploratory.

## 3. Results

**Figure 1:**
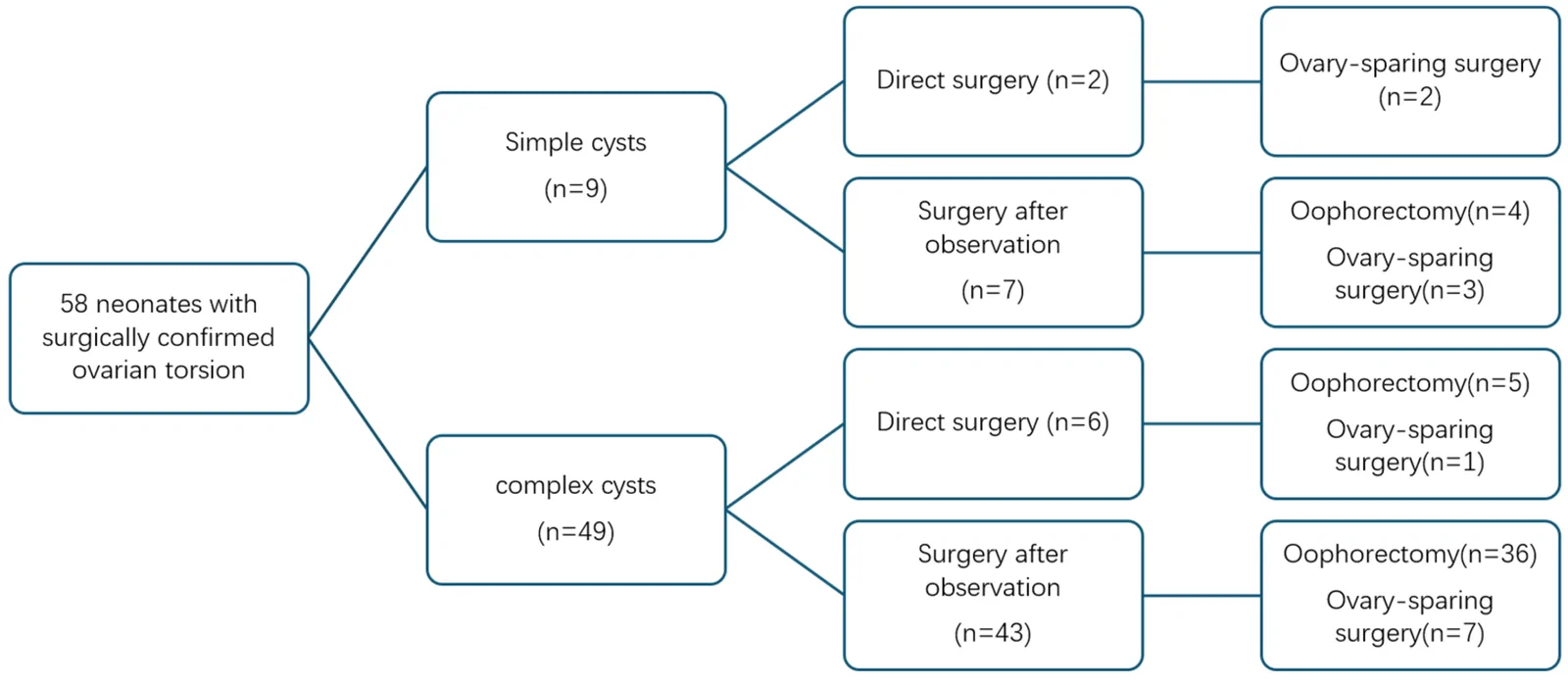
Management and outcomes of 58 neonates with ovarian torsion.

Fifty-eight infants with surgically confirmed ovarian cysts combined with torsion were enrolled in this study. Figure 1 illustrates their clinical courses, and Table 1 summarizes the baseline clinical characteristics. Median surgical age was 34 days (1–245 days). The majority of cases were diagnosed prenatally (94.8%, 55/58), and unilateral cysts accounted for 94.8% (55/58). Preoperative ultrasound revealed complex cysts in 84.5% (49/58) and simple cysts in 15.5% (9/58). Cysts with a maximum diameter ≥ 4 cm comprised 69.0% (40/58), while those <4 cm constituted 31.0% (18/58). Pathology identified two serous cystadenomas; the rest were non-neoplastic benign ovarian cysts. All neonates with prenatal ovarian cyst diagnosis remained asymptomatic at the time of referral. Among the three infants diagnosed after delivery, one was transferred to our center due to postnatal cyanosis. The other two sought medical care for gastrointestinal manifestations including vomiting and hematochezia.

Table 2 details the distribution of preoperative ultrasound features by surgical strategy. Overall cyst appearance differed between the oophorectomy and ovary-sparing groups (*p* = 0.020), with complex cysts accounting for 91.1% (41/45) versus 61.5% (8/13) (RD +29.6%, 95% CI: +1.9% to +57.3%). However, this did not retain significance after Bonferroni correction (adjusted α = 0.0026). Ultrasound-suspected torsion rates were 40.0% (18/45) versus 53.8% (7/13) (RD −13.9%, 95% CI: −44.5% to +16.8%; *p* = 0.526), and ultrasonographic signs of torsion with necrosis were comparable between groups (31.1% vs. 30.8%; RD +0.3%, 95% CI: −28.2% to +28.8%; *p* = 1.000). All seven individual sonographic features were similarly distributed (all *p* > 0.05). Overall, simple versus complex cyst classification showed the strongest numerical difference, though not significant after correction.

**Table 2:** Preoperative ultrasound features and surgical outcomes in 58 neonates with ovarian torsion.

Variable	Total (n = 58)	Oophorectomy Group (n = 45)	Ovary-Sparing Surgery Group (n = 13)	RD (95%CI)	*p* Value
Overall appearance					0.020
Complex	49 (84.5%)	41 (91.1%)	8 (61.5%)	+29.6% (+1.9% to +57.3%)	
Simple	9 (15.5%)	4 (8.9%)	5 (38.5%)		
Ultrasound suspicion of torsion	25 (43.1%)	18 (40.0%)	7 (53.8%)	−13.9% (−44.5% to +16.8%)	0.526
Torsion suspected only	7 (12.1%)	4 (8.9%)	3 (23.1%)	−14.2% (−38.6% to +10.2%)	0.180
Torsion with necrosis suspected	18 (31.0%)	14 (31.1%)	4 (30.8%)	+0.3% (−28.2% to +28.8%)	1.000
Individual sonographic features					
Internal low-level echoes	5 (8.6%)	5 (11.1%)	0 (0%)	+11.1% (−4.7% to +26.9%)	0.577
Fluid–fluid or fluid–debris level	16 (27.6%)	12 (26.7%)	4 (30.8%)	−4.1% (−32.3% to +24.1%)	0.739
Septation or reticular/mesh-like pattern	16 (27.6%)	15 (33.3%)	1 (7.7%)	+25.6% (+5.7% to +45.6%)	0.087
Internal floating echoes	3 (5.2%)	3 (6.7%)	0 (0%)	+6.7% (−8.3% to +21.6%)	1.000
Intracystic calcification	17 (29.3%)	15 (33.3%)	2 (15.4%)	+18.0% (−6.0% to +41.9%)	0.307
Thickened or echogenic wall	18 (31.0%)	15 (33.3%)	3 (23.1%)	+10.3% (−16.5% to +37.0%)	0.735
Solid component	15 (25.9%)	11 (24.4%)	4 (30.8%)	−6.3% (−34.4% to +21.7%)	0.724

Ultrasound suspicion of torsion: report explicitly diagnosed torsion or stated torsion could not be excluded. Torsion with necrosis suspected: report explicitly described torsion with necrosis.

In the 50 cases with serial ultrasound follow-up (Table 3), global comparisons of the ultrasound observation window, changes in cyst size, morphological shifts, and age at surgery between the oophorectomy and ovary-sparing groups were not statistically significant (all *p* > 0.05). The risk differences for each subgroup are presented with 95% confidence intervals, all crossing zero. Of note, the oophorectomy rate remained high among cases with cyst size reduction (11/12, 91.7%; RD +17.5%, 95% CI: −5.7% to +40.7%) and among those remaining complex on serial ultrasound (35/42, 83.3%; RD +17.5%, 95% CI: −12.7% to +47.7%). Only one case showed a simple-to-complex transition, precluding further analysis.

**Table 3:** Dynamic changes of ovarian cysts and surgical outcomes in the observation group (n = 50).

Variable	Total (N = 50)	Oophorectomy Group (n = 40)	Ovary-Sparing Surgery Group (n = 10)	RD (95%CI)	Global *p* Value
Ultrasound observation window					0.521
≤2 weeks	26 (52.0%)	21 (52.5%)	5 (50.0%)	+2.5% (−32.1% to +37.1%)	
2–12 weeks	19 (38.0%)	16 (40.0%)	3 (30.0%)	+10.0% (−22.2% to +42.2%)	
≥12 weeks	5 (10.0%)	3 (7.5%)	2 (20.0%)	−12.5% (−38.6% to +13.6%)	
Change in cyst size					0.331
Decreased (≥0.5 cm)	12 (24.0%)	11 (27.5%)	1 (10.0%)	+17.5% (−5.7% to +40.7%)	
Stable (within ±0.5 cm)	26 (52.0%)	21 (52.5%)	5 (50.0%)	+2.5% (−32.1% to +37.1%)	
Increased (≥0.5 cm)	12 (24.0%)	8 (20.0%)	4 (40.0%)	−20.0% (−52.8% to +12.8%)	
Change in cyst morphology					0.312
Complex → Complex	42 (84.0%)	35 (87.5%)	7 (70.0%)	+17.5% (−12.7% to +47.7%)	
Simple → Simple	7 (14.0%)	4 (10.0%)	3 (30.0%)	−20.0% (−49.9% to +9.9%)	
Simple → Complex	1 (2.0%)	1 (2.5%)	0 (0.0%)	+2.50% (−14.4% to +19.4%)	
Age at surgery					0.588
≤2 weeks	15 (30.0%)	12 (30.0%)	3 (30.0%)	+0.0% (−31.8% to +31.8%)	
2–12 weeks	25 (50.0%)	21 (52.5%)	4 (40.0%)	+12.5% (−21.6% to +46.6%)	
≥12 weeks	10 (20.0%)	7 (17.5%)	3 (30.0%)	−12.5% (−43.3% to +18.3%)	

Table 4 summarizes the surgical outcomes of preoperative simple and complex cysts. Among the 9 simple cysts, ovarian survival was confirmed on follow-up in 44.4% (4/9), compared with 0% (0/49) in the complex cyst group (RD +44.4%, 95% CI: +14.8% to +74.1%; global *p* < 0.001). Oophorectomy was performed in 44.4% (4/9) of simple cysts versus 83.7% (41/49) of complex cysts, with detailed surgical strategies presented in Table 4. Histological necrosis was observed in 44.4% (4/9) of simple cysts versus 98.0% (48/49) of complex cysts (RD −53.5%, 95% CI: −86.2% to −20.8%; *p* < 0.001). After Bonferroni correction, the difference in ovarian survival remained significant (adjusted α = 0.0026).

**Table 4:** Surgical management and outcomes in 58 patients with ovarian torsion.

Variable	Simple Cyst (n = 9)	Complex Cyst (n = 49)	RD (95%CI)	Global *p* Value
Preoperative ultrasound indicated torsion	2 (22.2%)	23 (46.9%)	−24.7% (−55.3% to +5.8%)	0.275
Therapeutic strategy				0.484
Detorsion + Fenestration	1 (11.1%)	0 (0%)	+11.1% (−12.0% to +34.2%)	
Detorsion + Cystectomy	4 (44.4%)	8 (16.3%)	+28.1% (−6.0% to +62.2%)	
Oophorectomy	4 (44.4%)	41 (83.7%)	−39.2% (−73.3% to −5.2%)	
Postoperative histopathology				0.001
No necrosis observed	4 (44.4%)	1 (2.0%)	+42.4% (+9.7% to +75.1%)	
Necrosis	4 (44.4%)	48 (98.0%)	−53.5% (−86.2% to −20.8%)	
Prognosis				
Ipsilateral ovarian survival	4 (44.4%)	0 (0%)	+44.4% (+14.8% to +74.1%)	<0.001

## 4. Discussion

Among the 58 surgically confirmed cases, ovarian survival was achieved in 44.4% (4/9) of preoperative simple cysts compared with 0% (0/49) of complex cysts (RD +44.4%, 95% CI: +14.8% to +74.1%; *p* < 0.001). Of note, among the 13 cases that underwent ovary-sparing surgery, only 4 (30.8%) had confirmed ovarian survival on follow-up ultrasound, meaning that nearly 70% of ovary-sparing procedures did not result in ovarian survival. In contrast, changes in cyst size or morphology during follow-up did not differ significantly between the oophorectomy and ovary-sparing surgery groups (all global *p* > 0.05). These findings suggest that preoperative ultrasound classification is associated with ovarian survival in neonatal ovarian torsion, and may inform preoperative counseling regarding the likelihood of ovarian salvage.

In our cohort, there were 9 cases of torsion that presented as “simple” on preoperative ultrasound, a subgroup that has been infrequently reported in the literature. In the surgical cases reported by Safa et al. [9], 3 cases were diagnosed with simple cysts, among which 1 case with a 69 mm cyst presented with torsion. In the study by Papic et al. [8], only 1 case had a simple cyst identified on prenatal ultrasonography; no change in cyst characteristics was noted after 7 weeks of observation (final diameter: 56 mm), and torsion was detected intraoperatively. Signorelli et al. [13] reported 12 fetal ovarian cyst cases that underwent surgical treatment, 4 of which were simple cysts and received postpartum surgery with a confirmed diagnosis of ovarian torsion; unfortunately, the specific prognostic outcomes were not mentioned in their report.

The ultrasonic classification system for neonatal ovarian cysts proposed by Nussbaum et al. [5] in 1988 has remained in use to date. A simplified consensus has gradually formed in clinical practice that simple cystic lesions can be managed with conservative observation. Among the 9 simple cysts, only 2 (22.2%) showed sonographic suspicion of torsion preoperatively, while the remaining 7 underwent surgery based on size-related indications (including one bilateral case operated for a contralateral giant cyst). This indicates that a simple appearance on preoperative ultrasound cannot reliably rule out ovarian torsion or necrosis. Short-term repeat ultrasound assessment by seasoned radiologists may be considered for simple cysts that persist or measure over 4 cm, even without detectable torsion manifestations. The presence of silent torsion in simple cysts suggests that closer sonographic assessment of ovarian position, vascularity, and the whirlpool sign—features originally described in broader pediatric populations—may be clinically useful, particularly when torsion is not overtly suspected [14,15,16].

Among the 49 torsion cases with preoperative complex cysts, the ipsilateral ovarian survival rate was 0% (0/49), and the histological necrosis rate was 98.0% (48/49). The presence of complex sonographic features (such as fluid-fluid levels, septations, or calcifications) signifies that hemorrhage, necrosis, or organization has taken place within the cyst, which is strongly associated with ovarian damage [12]. Surgical exploration in such cases is unlikely to achieve ovarian salvage [9]. These findings support clinicians in providing more realistic preoperative counseling to families regarding the likelihood of ovarian survival based on ultrasound appearance. Previous debates on the management of complex cysts have largely centered on whether surgery is indicated [17,18,19], without quantifying the probability of ovarian survival once torsion has occurred. While Tyraskis et al. [3] identified sonographic features associated with ovarian loss, the present study further provides quantitative estimates of ovarian salvage rates based on preoperative ultrasound appearance in surgically confirmed torsion cases.

In the 50 cases with serial ultrasound follow-up, none of the global comparisons for observation window, cyst size changes, or morphological shifts reached statistical significance between the oophorectomy and ovary-sparing surgery groups (all global *p* > 0.05; Table 3), and all corresponding 95% CIs for risk differences crossed zero. Nevertheless, among the 12 cases with cyst shrinkage, the oophorectomy rate remained high (11/12, 91.7%; RD +17.5%, 95% CI: −5.7% to +40.7%), suggesting that size reduction does not invariably indicate a benign resolution process. Given the small subgroup and wide confidence intervals, this observation is exploratory and hypothesis-generating rather than confirmatory. For cysts without clear sonographic evidence of torsion at initial assessment, a decrease in size on serial ultrasound should not be taken as definitive reassurance against torsion, though further validation is needed.

Pathological evaluation of the 58 surgical specimens revealed serous cystadenoma in two cases (3.4%), with all other lesions classified as benign non-neoplastic cysts. In both instances, preoperative sonography indicated torsion with necrotic changes, and neither patient experienced recurrence after surgery. Literature reports indicate that benign ovarian tumors account for about 2% of neonatal ovarian lesions, while malignant tumors remain exceedingly rare [2,20]. Serous cystadenoma belongs to benign tumors with slow growth rates; its prognosis remains unaffected even if surgery is deferred [21]. Given the scarcity of ovarian tumors in newborns, routine ultrasound surveillance provides adequate safety support for watchful waiting strategies.

Several methodological limitations should be considered when interpreting our findings. First, the statistical power of this study is constrained by its retrospective design and the modest cohort size. Second, we only included surgically confirmed torsion cases, excluding those successfully managed without operation; consequently, our results are directly applicable only to neonates with surgically proven ovarian torsion. Third, a single radiologist performed the ultrasound classification, and no formal interobserver reliability testing was conducted; thus, some degree of classification bias remains possible. Fourth, complex ultrasound features are largely a downstream marker of damage that has already occurred (e.g., hemorrhage or necrosis) rather than an independent prognostic variable measured before the outcome; therefore, our findings represent an association rather than a predictive relationship. Fifth, one simple cyst with no histological necrosis was not visualized on follow-up ultrasound, suggesting possible underestimation by sonography. Even reclassifying this case as survival, the rate for simple cysts (5/9, 55.6%; 95% CI: 26.7–81.1%) would still far exceed that for complex cysts (0/49, 0%), leaving our main conclusion unchanged. Sixth, ultrasound equipment and techniques may have changed during the decade-long study period; however, all images were interpreted by the same experienced radiologist, which likely mitigated the influence of such variability.

## 5. Conclusions

The overall ovarian survival rate after surgical intervention for neonatal ovarian torsion was modest (6.9%, 4/58), with an oophorectomy rate of 77.6% (45/58). Nevertheless, among surgically confirmed torsion cases, those with simple cysts on preoperative ultrasound had the highest ovarian survival rate (44.4%, 4/9), suggesting that this subgroup carries the best prognosis for ovarian salvage after surgical exploration. In contrast, none of the torsion cases with complex cystic sonographic features demonstrated ipsilateral ovarian survival on follow-up, suggesting that the potential for ovarian salvage is extremely limited in this subgroup.

## Figures and Tables

**Table 1:** Clinical features of 58 patients.

Variable	Data
Prenatal diagnosis	55 (94.8%)
Postnatal diagnosis	3 (5.2%)
Full-term	56 (96.6%)
Preterm	2 (3.4%)
Unilateral cyst	55 (94.8%)
Unilateral cyst + contralateral isolated torsion	1 (1.7%)
Bilateral cysts	2 (3.4%)
Preoperative ultrasound features	
Simple	9 (15.5%)
Complex	49 (84.5%)
Preoperative maximum diameter	
≥4 cm	40 (69.0%)
<4 cm	18 (31.0%)

## Data Availability

The data presented in this study are available on request from the corresponding author due to ethical restrictions.
